# Nanoformulation of Talazoparib Delays Tumor Progression and Ascites Formation in a Late Stage Cancer Model

**DOI:** 10.3389/fonc.2019.00353

**Published:** 2019-05-10

**Authors:** Paige Baldwin, Anders W. Ohman, Jamie E. Medina, Eric T. McCarthy, Daniela M. Dinulescu, Srinivas Sridhar

**Affiliations:** ^1^Department of Bioengineering, Northeastern University, Boston, MA, United States; ^2^Division of Women's and Perinatal Pathology, Department of Pathology, Brigham and Women's Hospital, Harvard Medical School, Boston, MA, United States; ^3^Department of Physics, Northeastern University, Boston, MA, United States; ^4^Division of Radiation Oncology, Harvard Medical School, Boston, MA, United States

**Keywords:** talazoparib, intraperitoneal therapy, nanoparticle, ovarian cancer, PARP inhibitor

## Abstract

Talazoparib, a potent PARP inhibitor, induces synthetic lethality in *BRCA*-deficient cancers making it an attractive candidate for ovarian cancer treatment. However, its potency lends itself to side effects associated more closely with traditional chemotherapeutics than other clinically approved PARP inhbitors. We sought to formulate Talazoparib in a nanoparticle delivery system, which allows the drug to be administered intraperitoneally. This was done to specifically target peritoneal dissemination of late stage metastatic ovarian cancer and increase talazoparib's therapeutic efficacy while minimizing toxic side effects. NanoTalazoparib was developed and characterized with regard to its size, loading, and surface charge. Talazoparib and NanoTalazoparib were tested on a panel of murine and human *BRCA* cell lines and the dose response was compared to Olaparib's, the currently used PARP inhibitor. Therapeutic efficacy was tested *in vivo* in a *Brca* peritoneal cancer model that mimics late stage disseminated disease. NanoTalazoparib has a diameter of about 70 nm with a neutral surface charge and ~75% encapsulation efficiency, which slowly releases the drug over several hours. Dose response analysis indicated that the murine cell lines with conditional *BRCA1/2, PTEN*, and *TP53* deletions had the lowest IC50s. NanoTalazoparib administered on a schedule of three doses weekly slowed disease progression and resulted in significantly less mice with ascites at the end point compared to controls. These results indicate that the slow release nanoformulation, NanoTalazoparib, effectively delivers PARP inhibitor therapy to the peritoneal cavity for disseminated cancer treatment. The ability to decrease ascites formation with the introduction of intraperitoneal NanoTalazoparib suggests this treatment may be an effective way to treat ovarian cancer-associated ascites and slow disease progression.

## Introduction

Ovarian cancer is the fifth leading cause of cancer mortality and the most lethal gynecological disease in women, with an estimated 14,000 deaths per year in the United States (1, 2). Two thirds of patients are diagnosed in the advanced stages of the disease when it is widespread and metastatic (3). This results in a 5-year survival rate of < 30%, which is mainly due to a late diagnosis and the development of chemoresistance associated with successive relapses (3, 4). This occurs due to tumors gradually adapting and developing resistance through genetic and epigenetic changes that are acquired during the course of repetitive chemotherapy cycles (5). Accordingly, there has been a recent focus on the development of novel therapies designed to strategically target specific pathways in the hopes of improving patient survival and quality of life. These targeted therapies, such as Poly(ADP-ribose) Polymerase (PARP) inhibitors, often exploit the concept of synthetic lethality (6, 7).

PARP inhibitors (PARPis) impair cells' ability to repair single strand DNA breaks via the base excision repair pathway, which results in double-strand breaks that cannot be repaired by cells with defective homologous recombination (HR) pathways (8). As 15% of ovarian cancer patients have mutations or inactivation of the *BRCA1* or *BRCA2* genes, which play a key role in double strand DNA break repair, and 50% of patients are thought to have defective HR pathways, these drugs are particularly effective for this disease (9–13). Talazoparib is the most potent of the PARPis to date, with superior efficacy compared to clinically approved Olaparib, due to its enhanced capability to trap PARP on the DNA and create cytotoxic lesions (14). Unfortunately, this enhanced potency is also associated with negative side effects more commonly seen with chemotherapeutics than other clinically approved PARPis (14–16). In a phase 3 clinical trial of talazoparib, 55% of patients experienced grade 3–4 hematologic adverse events, including anemia, thrombocytopenia, or neutropenia (17). Talazoparib is currently formulated for oral administration, which is easy to administer to patients. However, the bioavailability of Talazoparib in rats is only 56%, which means that the given dose must be higher in order to achieve a therapeutically relevant dose at the tumor site (18). One strategy for minimizing off-target side effects of drugs is to deliver them locally to the disease site (19).

In the case of ovarian cancer, intraperitoneal (i.p.) therapy, which targets the location of disseminated disease, was found to be more effective than intravenous (i.v.) treatment. A phase III clinical trial, GOG 172, found that i.p. therapy greatly enhanced both the median progression free survival and overall survival rate compared to i.v. therapy (20). However, patients in the i.p. therapy group had more side effects and a lower quality of life during and shortly after treatment. Consequently, better drug delivery systems need to be developed. To this end, nanotechnology-based vehicles have been engineered with an inherent ability to reduce toxicity while maintaining therapeutic efficacy (21). Nanoparticles injected in the peritoneal cavity are known to enter systemic circulation through the lymphatic system (22, 23). Furthermore, nanoparticle accumulation in the reticuloendothelial system and plasma is significantly lower for formulations administered i.p. vs. i.v. (24). Therefore, we sought to develop a system that would allow for the i.p. delivery of Talazoparib with the goal to increase therapeutic efficacy without compromising the quality of life. We hypothesized that a nanoformulation of Talazoparib would allow for a longer release of the drug delivered i.p. to the disease site, which could offer a therapeutic advantage over the current oral delivery method.

## Materials and Methods

### Synthesis of NanoTalazoparib

NanoTalazoparib was synthesized using 1, 2-dipalmitoyl-*sn*-glycero-3-phosphocholine (DPPC), 1,2-dioleoyl-3-tri methyl-ammonium-propane (chloride salt) (DOTAP), cholesterol, 1,2-distearoyl-*sn*-glycero-3 phosphoethanolamine-N-[methoxy(polyethyleneglycol)-2000 (DSPE-PEG_2000_, Avanti Polar Lipids), Talazoparib (Selleck Chemicals). DPPC, cholesterol, DOTAP and DSPE-PEG_2000_, were individually dissolved in chloroform and combined at a molar ratio of 65:29:2:4 with 65.7 mM Talazoparib in dimethyl sulfoxide. A thin film was formed by removal of solvents overnight on a rotary evaporator. The film was hydrated with phosphate buffered saline (PBS) pH 7.4 preheated to 50°C at a concentration of 6.1 mg lipid/mL PBS. Hydration was performed by incubating it in a 50°C water bath for 15 min followed by 1 min of vortex mixing. This cycle was repeated twice. Nanoparticles were sized by bath sonication for 20 min and allowed to rest overnight at 4°C. The non-encapsulated drug which is insoluble in aqueous media was removed via syringe filter (25). Vehicle nanoparticles (empty nanoparticles) were prepared following the same protocol without the addition of Talazoparib.

### Characterization of NanoTalazoparib

The size and zeta potential of the nanoparticles was measured using a Brookhaven 90Plus analyzer equipped with ZetaPALS. Nanoparticles were diluted 1:100 in PBS for all measurements. The size was confirmed by transmission electron microscopy using a negative stain of 1.5% phosphotungstic acid. The concentration of encapsulated Talazoparib was measured via high performance liquid chromatography (HPLC) following nanoparticle lysis with methanol. HPLC was performed on an Agilent 1260 Infinity II instrument with a reverse phase C18 Supelco column. The mobile phase was 50:50 methanol+0.1% trifluoroacetic acid (TFA): water+0.1% TFA with a flow rate of 0.4 mL/min. Talazoparib was detected at 311 nm and had a retention time of ~4.5 min.

### Drug Release Kinetics of NanoTalazoparib

Drug release kinetics was measured in a PBS bath at 37°C and pH 7.4 under constant stirring. Aliquots of the liposomal solution were removed at predetermined timepoints and lysed for HPLC analysis. Experiments were performed with 3 distinctly prepared batches of the formulation.

### Cell Culture

The murine fallopian tube (mFT) cell lines 3666, 3635, 3665, and 3707 used in this study were developed from conditional *Brca;Tp53;Pten* genetically engineered mouse models (GEMMs) of high-grade serous ovarian cancer (HGSOC) (26). Fallopian tubes collected from conditional GEMMs were cultured in a medium consisting of equal parts DMEM:F12 and M199 supplemented with HEPES pH 7.4 (10 mM), glutamine (2 mM), EGF (10 ng/mL), ITS-A (10 μg/mL), hydrocortisone (0.5 μg/mL), cholera toxin (25 ng/mL), retinoic acid (25 ng/mL), BSA (1.25 mg/mL), FBS (1% by volume), and transformed *in vitro* using 1 μg/mL doxycycline hyclate resuspended in media for 13 days (27, 28). The mFT cell lines were further transduced with a lentiviral vector to stably express the *luciferase (luc)* gene for use in bioluminescent assays *in vitro* and real time tumor imaging analysis *in vivo*. ASC34, ASC54, and ASC46 murine tumor lines were generated by culturing ascites collected from intraperitoneal murine tumor xenografts in the same cell culture medium detailed above.

The human HGSOC lines KURAMOCHI and OVSAHO were maintained in RPMI-1640 medium supplemented with 10% FBS and 1% penicillin streptomycin. JHOS2 was maintained in RPMI-1640 supplemented with 10% FBS, 1% penicillin streptomycin, and 1% non-essential amino acids. COV318 was maintained in DMEM supplemented with 10% FBS and 1% penicillin streptomycin. All cells were incubated at 37°C with 5% CO_2_.

### PARPi Dose Response Studies

Tumor cells were seeded at 500 or 1,000 cells/well in white microplates for *luc*-expressing lines or clear microplates for human HGSOC lines. In order to compare the efficacy of Talazoparib to a clinically approved PARP inhibitor, the dose response was compared to Olaparib and its nanoformulation, NanoOlaparib, which has been previously reported (29). Tumor cells were treated with Olaparib or NanoOlaparib at doses ranging from 10 nM to 100 μM and Talazoparib and NanoTalazoparib, respectively, at doses ranging from 250 pM to 10 μM for 6 days. The cellular viability of *luc*-expressing tumor murine lines was assayed in bioluminescence assays via addition of D-luciferin at a final concentration of 150 μg/ml. The cellular viability of human HGSOC lines was assayed in MTS assays. PARPi dose response graphs were plotted and fit using a variable slope four-parameter logistic equation constrained at 0 and 100 to determine the IC50 value (GraphPad Prism 7). All experiments were done in triplicate.

### Therapeutic Efficacy Comparison of PARPi and nanoPARPi Treatments *in vivo*

All animal studies and procedures were conducted in accordance with the Institutional Animal Care and Use Committee (IACUC) protocol #04187 reviewed and approved by the Harvard Medical Area Standing Committee on Animals.

NCr Nude *nu/nu* mice were purchased from Charles River Laboratories (Wilmington MA) and injected i.p. with 5 million 3666 cells in 500 μL PBS. All animals were imaged after 1 week to confirm engraftment and the successfully engrafted mice were separated into 4 groups: PBS vehicle (*n* = 5), empty nanoparticle vehicle (*n* = 5), oral Talazoparib (*n* = 9), and NanoTalazoparib (*n* = 9). Animals were treated 3 times weekly with 0.33 mg/kg NanoTalazoparib i.p. or 0.33 mg/kg Talazoparib via oral gavage. Oral Talazoparib was prepared by diluting a stock solution of Talazoparib with PBS pH 7.4. Both oral Talazoparib and NanoTalazoparib were prepared in 66 μg/mL solutions allowing for the delivery of a 5 μL/g body weight dose. Control groups were administered 5 μL/g bodyweight PBS or empty nanoparticles i.p., the volume equivalent of NanoTalazoparib. Tumor progression was monitored weekly via bioluminescence imaging following administration of 150 mg/kg luciferin injected i.p. using an IVIS Lumina II system (PerkinElmer, Waltham MA). Mice were observed daily for development of peritoneal ascites fluid. The first sign of ascites fluid was logged when the ventral abdomen of the mouse began to darken due to peritoneal bloody fluid accumulation. Mice were euthanized in accordance to established humane endpoint criteria, including the inability to ambulate, low body condition score, tumors in excess of 10% body weight, and dyspnea related to fluid accumulation. Animals were treated for 8 weeks and euthanized 72 h after the final dose for uniform quantification of final tumor burden. All tumors collected from each animal were weighed collectively to determine the total tumor weight per animal; in addition, tumor ascites were collected and their volume carefully measured and recorded. Animals that presented with peritoneal bloody fluid >200 μL at the time of necropsy, that had not previously demonstrated visual signs of ascites, were recorded as having developed ascites at the endpoint (day 57) (30). The tumor growth inhibition was assessed based on the aggregate tumor weight measurements and calculated using the formula shown below.

$$\begin{array}{l} \text{Tumor growth inhibition }(\%\text{ TGI})\\ \;=\left(1-\frac{mean\;tumor\;weight\;in\;treated\;group}{mean\;tumor\;weight\;in\;control\;group}\right)x\;100 \end{array}$$

### Histology

Tumors, liver, lungs, heart, kidneys, and spleen were harvested during necropsy and fixed in 10% formalin prior to blocking in paraffin. Slices of the organs and tumors were stained with hematoxylin and eosin (H&E).

### Tumor Characterization

#### Immunohistochemistry

PAX8 immunohistochemical staining of the mFT cell line 3666 and a tumor derived from this xenograft (T#58, Control) was performed on a Leica Bond automated staining platform using the Leica Biosystems Refine Detection Kit with citrate antigen retrieval. The PAX8 polyclonal antibody (Proteintech #10336-1-AP) used for immunohistochemical staining was diluted 1:600.

#### Cre-mediated Recombination

Cre-mediated recombination of *Brca2, Tp53*, and *Pten* in tumor tissue was detected by PCR according to published methods (26). DNA was isolated from tumor samples using a DNA Isolation Kit (DNeasy Blood & Tissue Kit 50, 69504, Qiagen). PCR was performed using GoTaq Green Taq Master Mix (Promega, PAM7122) and targeted primer pairs to confirm Cre-mediated recombination events in all tumor samples. The *Brca2* recombination reaction was carried out using 1 μg DNA for most tumor samples with the exception of T #49 (1.5 μg) and T#58 (4 μg). The *Pten* and *Tp53* recombination reactions used 200 ng DNA per tumor sample. The *Tp53 mutant* recombination reaction used 500 ng DNA for all samples except T#34, T#36, T#37, and T#47 which used 200 ng DNA, and T#41 and T#46 which used 700 ng DNA. Primer sequences and PCR programs can be found in Supplementary Methods.

### Toxicity Assessment

Swiss Webster mice were purchased from Charles River Labs (Wilmington, MA). Two days prior to treatment 150 μL of blood was collected via retro-orbital bleed in EDTA tubes for complete blood count analysis. Animals were separated into groups and treated with either 0.33 mg/kg talazoparib by oral gavage (*n* = 11) or NanoTalazoparib i.p. (*n* = 11). Animals were treated every other day for a total of 3 treatments. Twenty four hours after the final treatment animals were euthanized and blood was collected via cardiac puncture. Two hundred μL of blood was collected in EDTA tubes for complete blood count and the rest was allowed to clot and centrifuged to isolate the serum. Serum was assessed for alanine transaminase (ALT), aspartate transaminase (AST), lactate dehydrogenase (LDH), and creatinine. Blood was also collected from 5 animals that had undergone no treatment to assess baseline enzyme levels. All samples were immediately sent for analysis to VRL (Gaithersburg, MD) after collection.

### Statistical Analysis

All *in vitro* data were plotted as mean ± SD. The statistical significance of *in vitro* data was determined by using one-way ANOVA followed by Tukey's test for significance or Student's *t*-tests with α = 0.05. All *in vivo* data were plotted as mean ± SEM. PBS and empty nanoparticle controls were grouped together as average tumor progression in both subgroups was comparable. *In vivo* efficacy data normality was tested with the D'Agostino-Pearson test and *p* < 0.05 not considered a normal distribution. Significance of data not following a normal distribution was assessed via Kruskal-Wallis ANOVA followed by Dunn's test for multiple comparisons with α = 0.05. Significance of normal data was tested with one-way ANOVA followed by Tukey's test for significance or Student's *t*-tests with α = 0.05. For the proportion of animals that developed ascites a Chi-square test was used to compare proportions. The statistical significance of toxicity results was determined using one-way ANOVA followed by Tukey's test to compare treatments to controls. All statistical testing computed with Prism 7.

## Results

### Characterization of NanoTalazoparib

**Figure 1:**
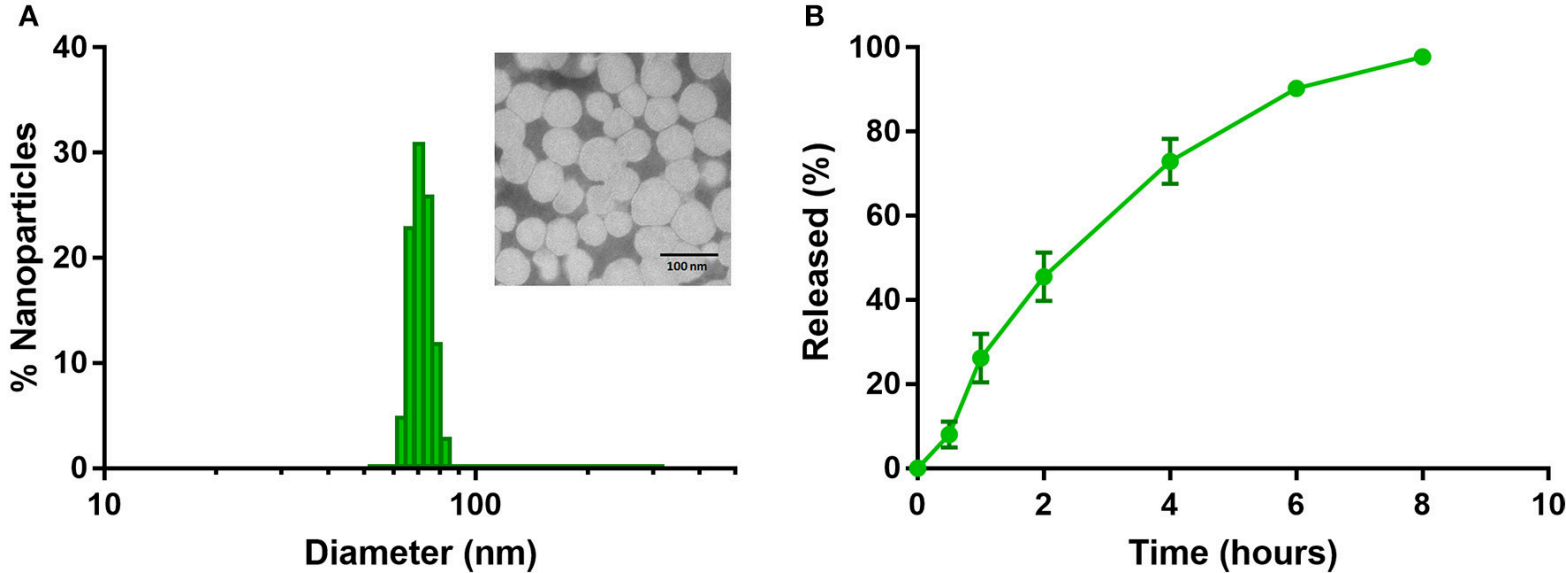
**(A)** Physicochemical characterization of NanoTalazoparib measured by dynamic light scattering and (inset) transmission electron microscopy after staining with 1.5% phosphotungstic acid. **(B)** Drug release kinetics of Talazoparib from nanoparticles at 37°C under constant agitation.

Dynamic Light Scattering measurements indicate NanoTalazoparib has a number weighted average diameter of 71.4 ± 12.0 nm, with a small population of particles ~200 nm (Figure 1A). The polydispersity index of 0.224 ± 0.009 signals the second, small, population of particles. Transmission electron microscopy confirmed that most of the particles are ~70 nm in diameter (Figure 1, inset). The zeta potential of these particles is near neutral at 3.98 ± 2.3 mV indicating the DSPE-PEG has created a shell that is proficient at shielding the positively charged DOTAP. The encapsulation efficiency of the particles is 76.9 ± 11.35% yielding therapeutically relevant concentrations of 153.8 ± 22.7 μg Talazoparib/mL. NanoTalazoparib has first-order release kinetics and releases over the course of 8 h in sink conditions at 37°C under constant agitation (Figure 1B).

### PARPi Dose Response Studies

**Figure 2:**
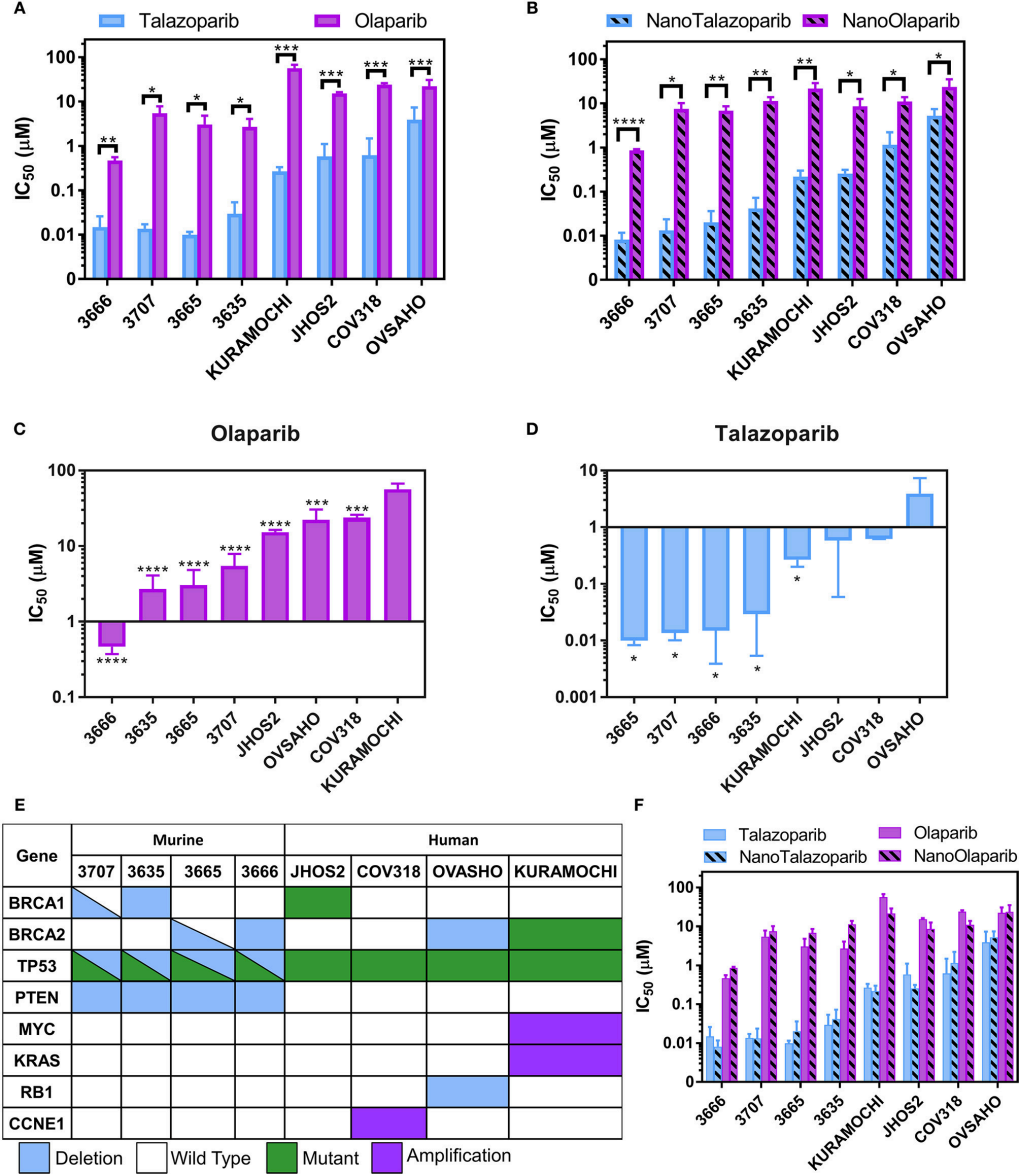
IC50 values for a panel of 4 murine FT lines and 4 human HGSOC lines treated with **(A)** free Olaparib or Talazoparib and **(B)** NanoOlaparib and NanoTalazoparib. KURAMOCHI was the least sensitive line to Olaparib **(C)** while OVSAHO was the least sensitive to Talazoparib **(D)**. Description of **(E)** mutations and deletions of interest in the 8 cell lines tested. Comparison of all treatments in all cell lines **(F)** reveals no difference in free vs. nanoformulations. All experiments were performed in triplicate. Statistical significance between two treatments in a single line was tested with Student's *t*-tests. Significance between lines was analyzed using one-way ANOVA followed by Tukey's test for significance, ^*^p < 0.05, ^**^p < 0.01, ^***^p < 0.001, ^****^p < 0.0001.

Talazoparib and NanoTalazoparib were more potent in all cell lines compared to Olaparib and NanoOlaparib, with a 10-fold decrease in IC50 value for the least sensitive lines (Figures 2A,B). KURAMOCHI was least sensitive to Olaparib treatment compared to all the other cell lines but was more sensitive to Talazoparib than OVSAHO (Figures 2C,D). All murine mFT cell lines (3666, 3665, 3635, and 3707) established from *Brca/Tp53/Pten* GEMMs showed superior sensitivity to PARPi treatment compared to the human cell lines despite homologous recombination defects being present in nearly all lines tested (Figure 2E). There was no significant difference in drug sensitivity between free drug and nanoformulation treatments, suggesting the drug is as active when released from the nanoparticles as it is in its free form Figure 2F.

### NanoTalazoparib Delays the Formation of Tumor Ascites

**Figure 3:**
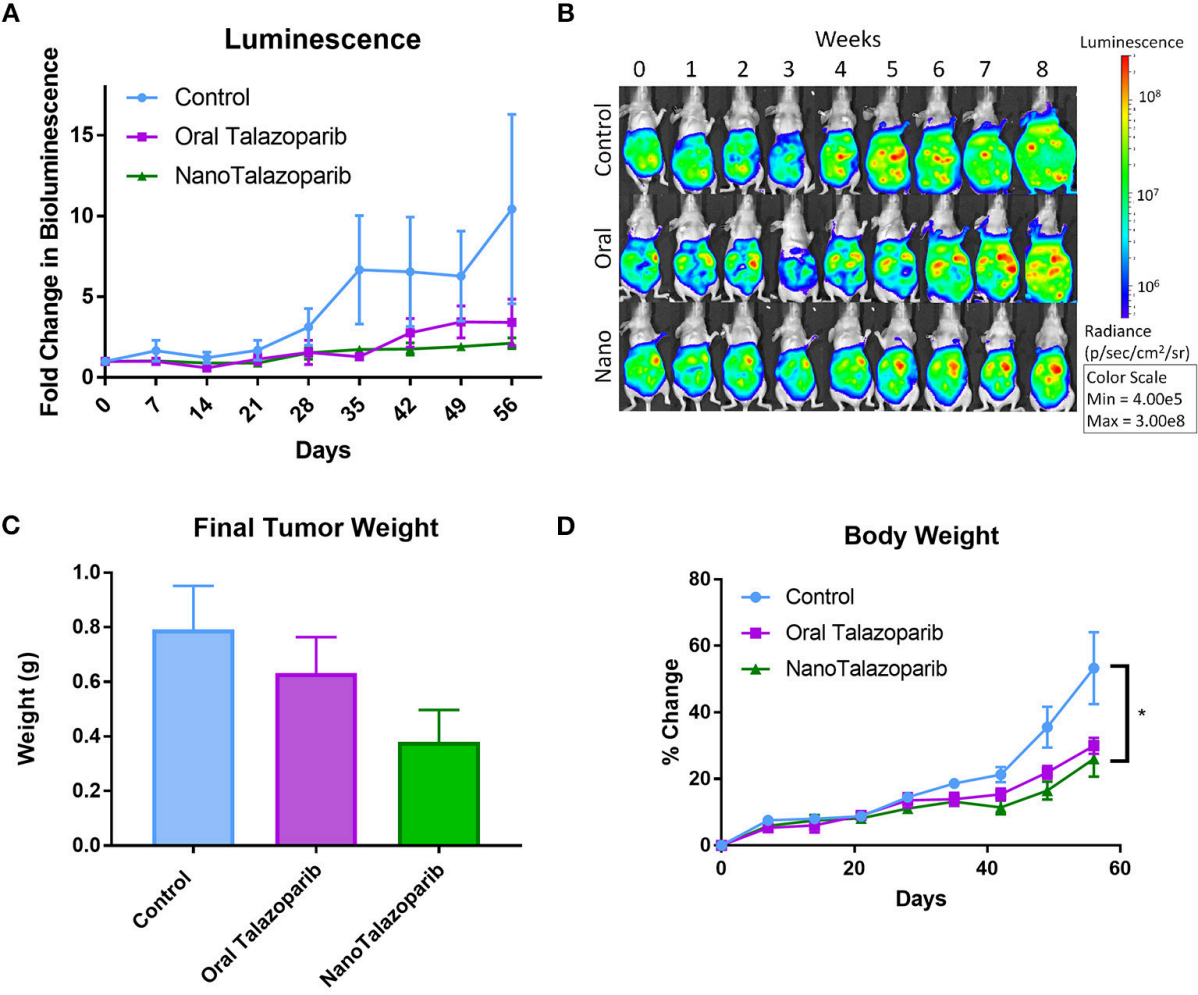
**(A)** Bioluminescence imaging allows for monitoring disease progression throughout the treatment period (*n* = 10 for control and *n* = 9 for treated groups). **(B)** Luminescence over time is displayed in representative images for each cohort throughout the course of the study. **(C)** The aggregate tumor weight for each animal at the end of the 8-week treatment period confirms final tumor burden (*n* = 8 for control and *n* = 9 for treated groups). **(D)** No evidence of gross toxicity was observed as measured by the percent change in bodyweight (*n* = 10 in control group and *n* = 9 in treated groups). Statistical significance was determined using two way ANOVA followed by Tukey's test, ^*^p < 0.05.

Tumor imaging studies indicated that the disease was progressing in control animals treated with PBS or empty nanoparticle vehicle as their tumor luminescence increased by a factor of 1.8 by week 3 (Figures 3A,B). Oral Talazoparib-treated animals began to progress by week 6 with an average fold change in luminescence of 2.9. NanoTalazoparib-treated animals began to progress by week 8, with an average fold change in luminescence of 2.1. In order to corroborate the bioluminescence data, animals were sacrificed 72 h after the final treatment to quantify final tumor weights and volume of ascites. Two animals in the control group were found dead on the day of sacrifice and tumors were unable to be collected from these animals. As expected, the control animals had the greatest tumor burden followed by the oral Talazoparib and NanoTalazoparib-treated groups, respectively, though the difference did not reach statistical significance (Figures 3B,C). Administration of Talazoparib via the nanoformulation increased the percent tumor growth inhibition from 34% in the oral group to 64% in the NanoTalazoparib group. Body weight measurements, which were used to monitor gross toxicity throughout treatment, indicated that all treatments were well-tolerated, however, with the buildup of ascites fluid the increased body weight also reflected the presence of ascites (Figure 3D, ^*^p < 0.05).

**Figure 4:**
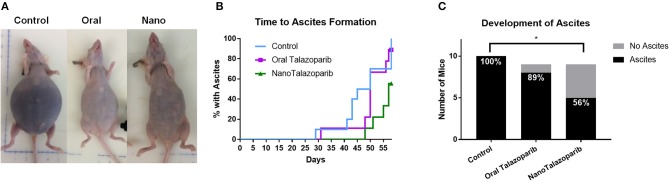
**(A)** The accumulation of tumor ascites and abdominal distension are shown in representative images from each cohort at the time of sacrifice. **(B)** Mice were carefully monitored and the day in which each animal presented with ascites was recorded (*n* = 10 in control group and *n* = 9 in treated groups). **(C)** Number of mice with ascites at the endpoint. Statistical significance was determined using Chi-square for proportions, ^*^p < 0.05 vs. control.

The formation of tumor ascites was closely monitored in controls and all treated animals (Figure 4A). Treatment with NanoTalazoparib prolonged the first signs of ascites development to 48 days from 29 and 31 days in the control and oral Talazoparib groups, respectively (Figure 4B). The median time to formation of ascites was 45, 50, and 57 days for control, oral Talazoparib, and NanoTalazoparib, respectively (Figure 4B). At the time of sacrifice, all 10 control mice (100%) and 8/9 (89%) oral Talazoparib-treated animals had developed ascites compared to only 5/9 (56%) of NanoTalazoparib-treated animals (Figure 4C). NanoTalazoparib treatment significantly decreased the proportion of animals that presented with ascites at the endpoint compared to controls (Figure 4C, ^*^p < 0.05). Supplementary Figures 1–3 present all of the mice at the endpoint with the volume of ascites collected.

### Assesment of Drug Toxicity

**Figure 5:**
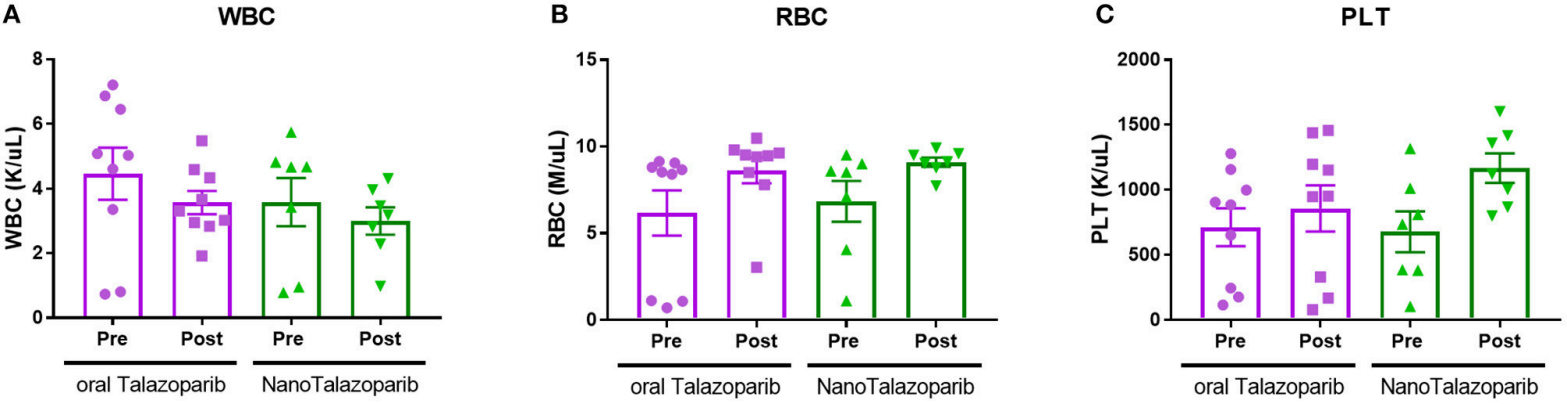
Changes in **(A)** WBC, **(B)** RBC, and **(C)** PLT counts following treatment with 3 doses of either oral Talazoparib or NanoTalazoparib (*n* = 9 for oral and *n* = 7 for nano).

**Figure 6:**
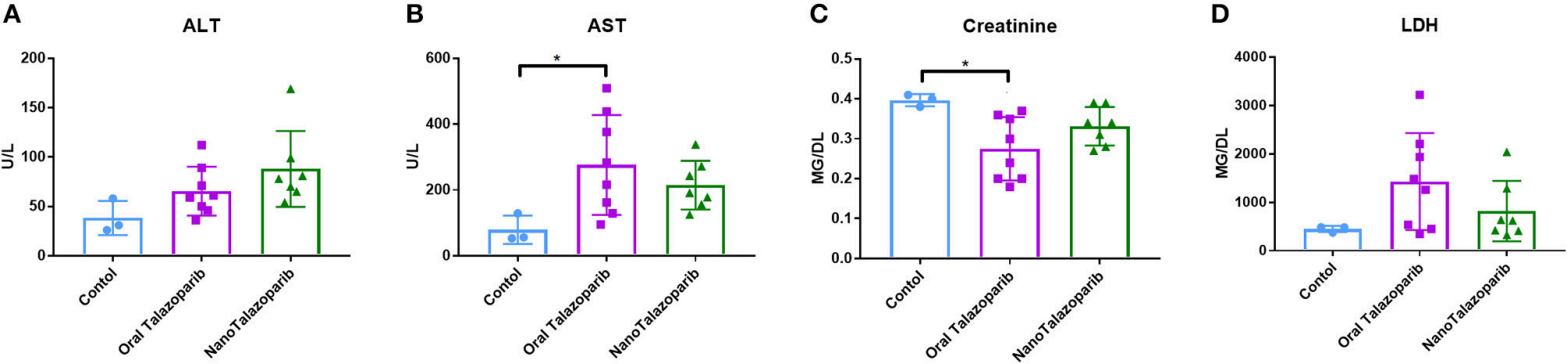
Comparison of serum levels of **(A)** ALT, **(B)** AST, **(C)** Creatinine, and **(D)** LDH following no treatment (*n* = 3) or treatment with 3 doses of either oral Talazoparib (*n* = 7) or NanoTalazoparib (*n* = 8); Statistical significance was determined using one way ANOVA followed by Tukey's test, ^*^p < 0.05 vs. control.

**Figure 7:**
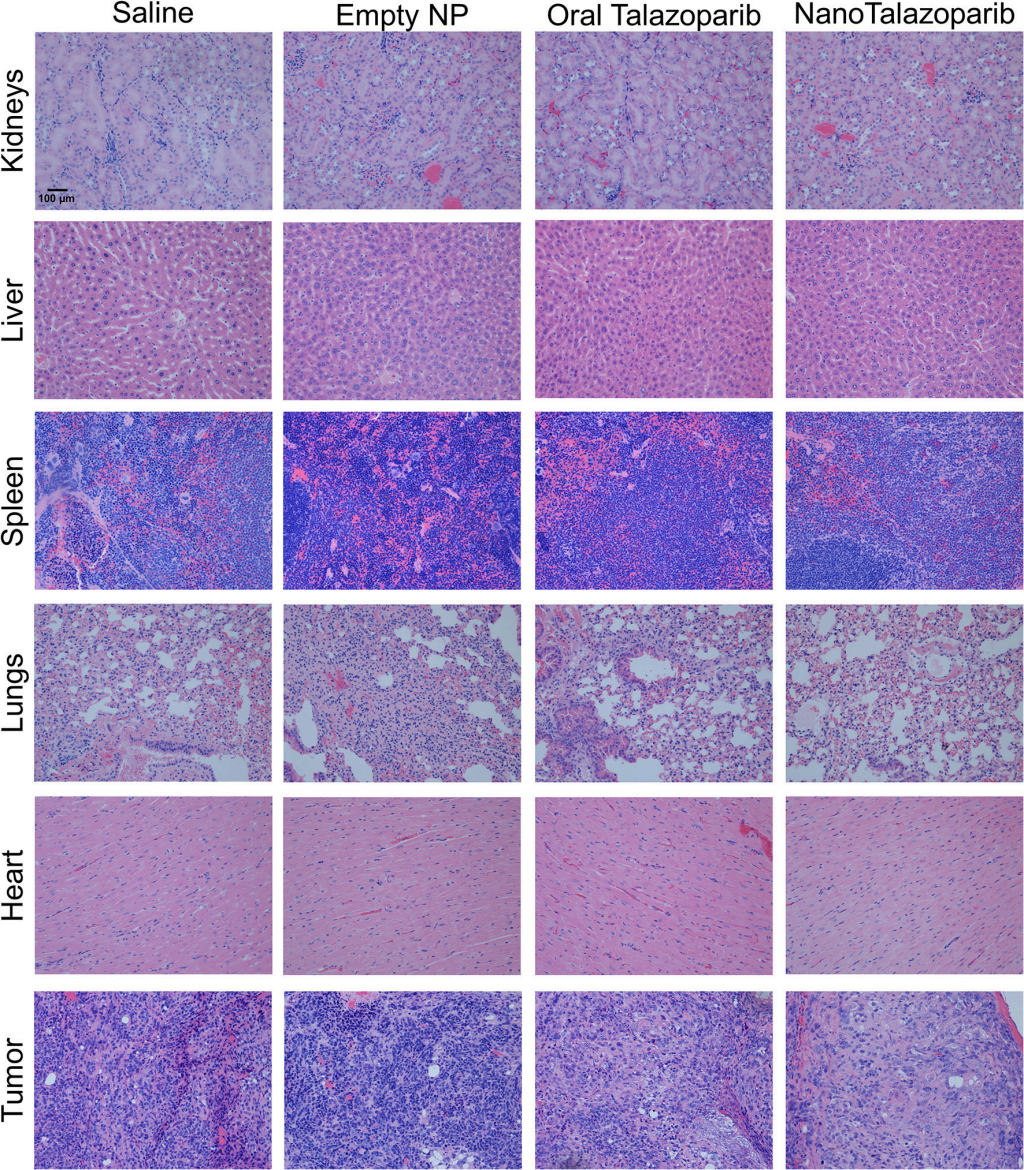
Representative slices of organs and tumors stained with hematoxylin and & eosin demonstrate no treatment associated toxicities in any groups after 8 weeks of continuous treatment. 20× magnification.

The effect of the different treatment modalities on blood cells and serum toxicity markers was assessed 24 h after 1 week of treatment (3 doses). On average there was no decrease in white blood cell (WBC), red blood cell (RBC), or platelet (PLT) counts after treatment with either oral Talazoparib or NanoTalazoparib (Figure 5). The fold change for each cell type was assessed using paired samples from before and after treatment and levels below 50% of the initial value were considered to have decreased. Based on paired samples, 33% (3/9) of animals treated with oral Talazoparib had decreased WBC levels compared with 14.2% (1/7) of animals treated with NanoTalazoparib but the difference did not reach statistical significance. Twenty two percent (2/9) of mice in the oral Talazoparib group had decreased RBC and PLT counts while no animals in the NanoTalazoparib group experienced RBC or PLT decreases. AST levels were significantly increased in oral Talazoparib treated animals compared to untreated animals while no statistically significant difference was seen between NanoTalazoparib-treated mice and controls (Figure 6, ^*^p < 0.05). In addition, creatinine levels were significantly lower in animals treated with oral Talazoparib compared to controls while no statistically significant difference was seen between NanoTalazoparib-treated mice and controls (Figure 6, ^*^p < 0.05). No statistical differences were reached when analyzing ALT and LDH levels between groups (Figure 6). Mice treated with NanoTalazoparib long term showed no signs of obvious damage to the organs of the mononuclear phagocyte system, including the kidneys, liver, spleen, lungs, and heart (Figure 7). These results were confirmed via histopathological analysis by a trained rodent pathologist.

### Tumor Characterization

Cre-mediated recombination for *Brca2, Tp53*, and *Pten* was validated in the parental mFT tumor lines (3666) used for tumor implantation studies and tumor xenografts (Supplementary Figure 4A). In order to confirm that the cell line and tumors were composed of fallopian tube secretory cells derived from the HGSOC mouse model (25), PAX8 marker staining was performed on the 3666 cell line as well as sectioned tumor from control xenograft #58 (Supplementary Figures 4B,C).

### Post Treatment Sensitivity of Tumor Ascites

**Figure 8:**
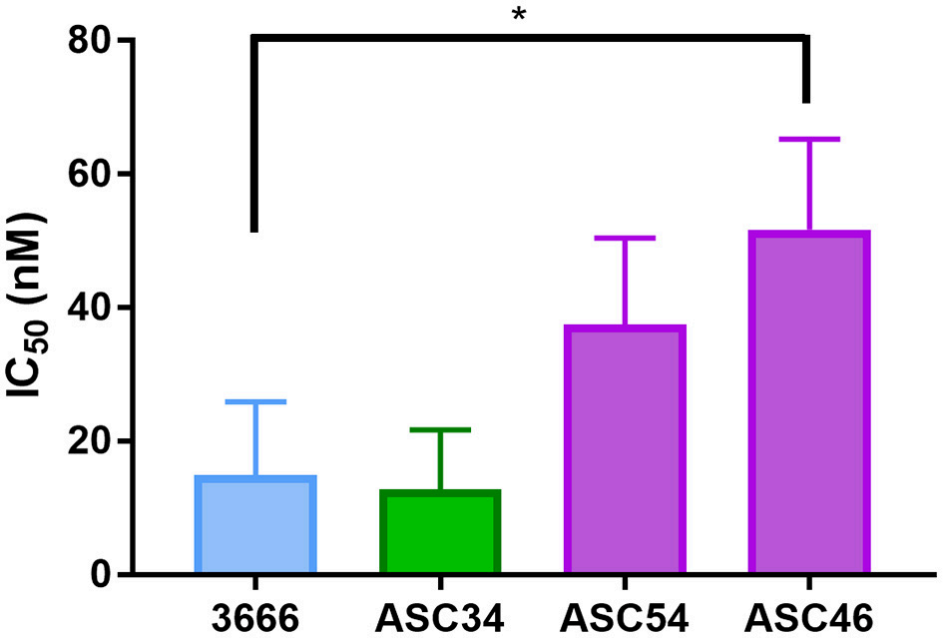
Comparison of IC50 values for tumor ascites lines and the parental mFT line 3666. ASC34 was derived from an animal treated with NanoTalazoparib that developed ascites at 48 days. ASC54 and ASC46 were derived from animals treated with oral Talazoparib and developed ascites after 57 and 50 days of treatment, respectively. All experiments performed in triplicate. Statistical significance determined using one way ANOVA followed by Tukey's test compared to 3666, ^*^p < 0.05.

Cell lines were generated from tumor ascites in order to assess whether or not Talazoparib-treated animals had developed PARPi-resistant disease. One line (ASC34) was developed from an animal treated with NanoTalazoparib while the other two lines (ASC54 and ASC46) were generated from animals treated with oral Talazoparib (Table 1). These cell lines were treated with various doses of free Talazoparib in order to determine the IC50 values post *in vivo* treatment. ASC34 had a similar IC50 to the parental tumor line, mFT 3666 (Figure 8). ASC54 and ASC46 showed slightly higher IC50 values than 3666, however, only ASC46 was statistically higher (Figure 8, ^*^p < 0.05).

**Table 1:** Overview of cell lines derived from tumor ascites and corresponding IC50 values.

**Cell line**	**Treatment**	**Days to ascites**	**IC50**
3666	Parental line; treatment naïve		14.9 ± 11.0
ASC34	NanoTLZ	48	12.8 ± 9.0
ASC54	Oral TLZ	57	37.5 ± 7.5
ASC46	Oral TLZ	50	51.7 ± 13.5

## Discussion

The development of an injectable formulation of Talazoparib has the potential to bypass some of the limitations associated with the current oral administration and provide a means for i.v. or i.p. therapy. In this work, we present a nanoformulation of Talazoparib, which can be administered directly into the peritoneal cavity as a means of targeting the treatment to the site of disseminated disease. Dose response analysis of Talazoparib, Olaparib, and the respective nanoformulations confirmed that Talazoparib is more potent than Olaparib not only in the mFT model of interest but also in all human HGSOC lines tested (Figures 2A,B). The mFT cell lines had lower IC50 values for all treatments, with 3666 displaying the lowest values overall. This is likely due to these lines having deficiency in both *BRCA* and *PTEN*, which is linked to genome stability and PARPi sensitivity (31). No significant differences were observed between the IC50 values of the mFT lines. JHOS2, OVSAHO, and KURAMOCHI all harbor mutations or deletions in either *BRCA1* or *BRCA2*, rendering them HR-deficient. However, COV318, the only line in the screen which is HR proficient and therefore, should not be sensitive, is more sensitive than KURAMOCHI to Olaparib (^***^p < 0.001) and though not significant, has a lower IC50 value for Talazoparib than OVSAHO. This may be a function of the experimental design, as single agent efficacy is dependent on replication and all lines were treated for the same length of time while OVSAHO has a longer doubling time than the other lines (data not shown). The sensitivity profiles varied between the two drugs as KURAMOCHI was the least sensitive to Olaparib but more sensitive to Talazoparib than OVASHO. It has been previously shown that Talazoparib is approximately 100 times more effective at trapping PARP-DNA complexes than Olaparib; however, the capacity to inhibit PAR synthesis is only about 1.5 times better (32). The ability of Talazoparib to sensitize cells such as KURAMOCHI that are resistant to Olaparib, but not cells such as OVSAHO, suggests differential PARP trapping amongst different cell lines, perhaps due to variances in basal PARP1 levels.

When administered 3 times weekly for 8 weeks NanoTalazoparib, resulted in an average tumor growth inhibition of 64%, however, there is no statistical significance between control and NanoTalazoparib final tumor weight. We hypothesized that NanoTalazoparib would be more effective than the oral form, as the bioavailability of the oral drug is 56% in rats while the nanoformulation is delivered directly to the disease site and available peritoneally where the disseminated tumor cells are present (18). It has been previously shown that nanoparticles administered into the peritoneal cavity are cleared via the lymphatic system and enter systemic circulation (22, 33). Studies show that only 10% of nanoparticles with similar properties to NanoTalazoparib remain in the peritoneal cavity 24 h after injection (34). NanoTalazoparib is released slowly over the course of 8 h in order to prevent flooding the cavity with the entire dose at one time.

In a phase 3 clinical trial using oral Talazoparib 52.8% of patients presented with anemia, 26.9% with thrombocytopenia, and 17.1 % with leukopenia (17). Our results showed that 33% of animals in the oral Talazoparib group had at least a 50% decrease in WBC counts after treatment and 22% presented with at least a 50% decrease in RBC and PLT counts, although the difference did not reach statistical significance between groups. In contrast, only 14% of animals in the NanoTalazoparib had a 50% decrease in WBC counts and no animals presented with RBC or PLT counts < 50% of baseline, although the difference did not reach statistical significance between groups. In regards to enzyme levels, AST levels were significantly increased in oral Talazoparib treated animals compared to untreated animals while no statistically significant difference was seen between NanoTalazoparib-treated mice and controls. In addition, creatinine levels were significantly lower in animals treated with oral Talazoparib compared to controls while no statistically significant difference was seen between NanoTalazoparib-treated mice and controls. No statistical differences were reached when analyzing ALT and LDH levels between groups.

The reduced proportion of mice treated with NanoTalazoparib presenting with ascites at the endpoint demonstrates the potential ability of the i.p. delivery of the nanoformulation to delay the formation of ascites. More than one-third of women diagnosed with ovarian cancer will develop ascites from the disease (35, 36). Typically, treatment of the underlying disease will resolve the ascites but the development of chemoresistant disease results in intractable ascites. Ascites buildup has been shown to be a result of increased fluid production from both the tumor cells and tumor free peritoneum combined with compromised draining due to obstructed lymphatics (37, 38). VEGF has been shown to play a role in formation of malignant ascites by increasing vascular permeability, and studies have shown inhibition of VEGF can prevent ascites accumulation (39–41). Studies have indicated PARP1 is plays a role in angiogenesis and can decrease VEGF expression (42–44). Inhibition of PARP1 and PARP1 knockouts have shown a decrease in induction of the transcription factor HIF-1α, which upregulates VEGF expression (45, 46). It would be interesting to test in the future whether the i.p. delivery of NanoTalazoparib could potentially decrease VEGF expression in the peritoneum and subsequently decrease the production of fluid.

Notably, several of the cell lines derived from ascites appear to be sensitive to Talazoparib at least when tested *ex vivo* in 2D cell cultures. ASC46 has a significantly higher IC50 value than the parental tumor line (mFT 3666) but would still be considered PARPi sensitive (47). There does not appear to be a correlation between when the animals began to develop ascites, the subsequent final ascites volume, and drug sensitivity. Of the three ascites lines generated, ASC34 was derived from the animal that developed signs of ascites earlier than the others, however, it was the most sensitive line to the rechallenge with Talazoparib. Future studies could probe ascites sensitivity at various time points following the delivery of oral or i.p. nanoformulation in order to elucidate the effect of the treatment route on the development of PARPi resistant disease. It would be interesting to further determine whether NanoTalazoparib could be used as a maintenance therapy or in combination with other therapies to potentially slow down the disease progression and delay the formation of ascites.

## Ethics Statement

This study was carried out in accordance with the recommendations of the Institutional Animal Care and Use Committee (IACUC) protocol #04187 reviewed and approved by the Harvard Medical Area Standing Committee on Animals.

## Author Contributions

PB, AO, DD, and SS designed research. PB and AO performed research. PB and SS contributed new reagents. AO, JM, and EM generated and characterized murine cell lines and mouse models. PB and AO analyzed data and wrote the manuscript. DD and SS edited the manuscript.

### Conflict of Interest Statement

The authors declare that the research was conducted in the absence of any commercial or financial relationships that could be construed as a potential conflict of interest.
