# Supplementary material for: Nanoformulation of Talazoparib Delays Tumor Progression and Ascites Formation in a Late Stage Cancer Model
Source: Front Oncol. 2019 May 10;9:353. doi: 10.3389/fonc.2019.00353 (PMC6524318; doi:10.3389/fonc.2019.00353)
Supplement: Supplementary file 1 [file Data_Sheet_1.pdf]

Supplementary Material

1 Supplementary Methods

Primer sequences for PCR are shown below:

Brca2: Cre-mediated recombination forward: 5'- GGC TGT CTT AGA ACT TAG GCT G-3'; Cre-mediated recombination reverse: 5'- TGT TGG ATA CAA GGC ATG TAC AC-3'

Tp53: Cre-mediated recombination forward: 5'- CAC AAA AAC AGG TTA AAC CCA G-3'; Cre-mediated recombination reverse: 5'- GAA GAC AGA AAA GGG GAG GG-3'

Tp53 mutant: Cre-mediated recombination forward: 5'- AGC CTG CCT AGC TTC CTC AGG-3'; Cre-mediated recombination reverse: 5'- CTT GGA GAC ATA GCC ACA CTG-3'

Pten: Cre-mediated recombination forward: 5'- ACT CAA GGC AGG GAT GAG C-3'; Cre-mediated recombination reverse: 5'- GCT TGA TAT CGA ATT CCT GCA GC-3'

PCR Reaction conditions were as follows:

The PCR reaction for Cre-mediated *Brca2* recombination consisted of incubation at 94°C for 3 minutes followed by 35 cycles of 94°C for 1 minute, 60°C for 2 minutes, and 72°C for 3 minutes, followed by 3 minutes at 72°C and finally 12°C indefinitely.

The PCR reaction for Cre-mediated *Tp53* recombination and Cre-mediated *Tp53* mutant recombination consisted of incubation at 94°C for 2 minutes followed by 29 cycles of 94°C for 30 seconds, 58°C for 30 seconds, and 72°C for 50 seconds, followed by 5 minutes at 72°C and finally 12°C indefinitely.

The PCR reaction for Cre-mediated *Pten* recombination consisted of incubation at 94°C for 2 minutes followed by 35 cycles of 94°C for 30 seconds, 58°C for 1 minute, and 72°C for 1 minute 10 seconds, followed by 10 minutes at 72°C and finally 12°C indefinitely.

2 Supplementary Figures

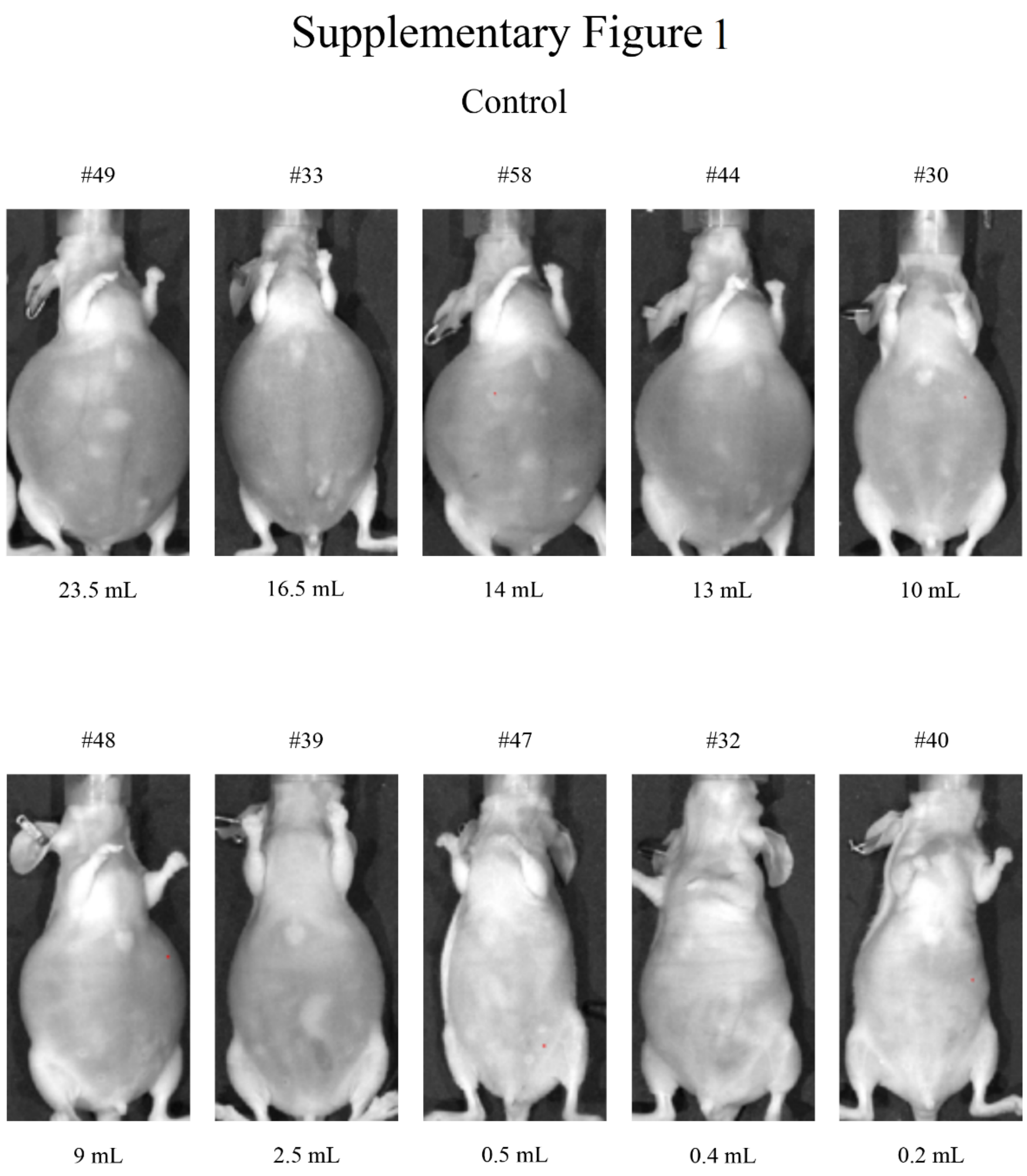

Supplementary Figure 1. Vehicle treated mice at the endpoint and volume of collectable ascites fluid.

Supplementary Figure 2

Oral Talazoparib

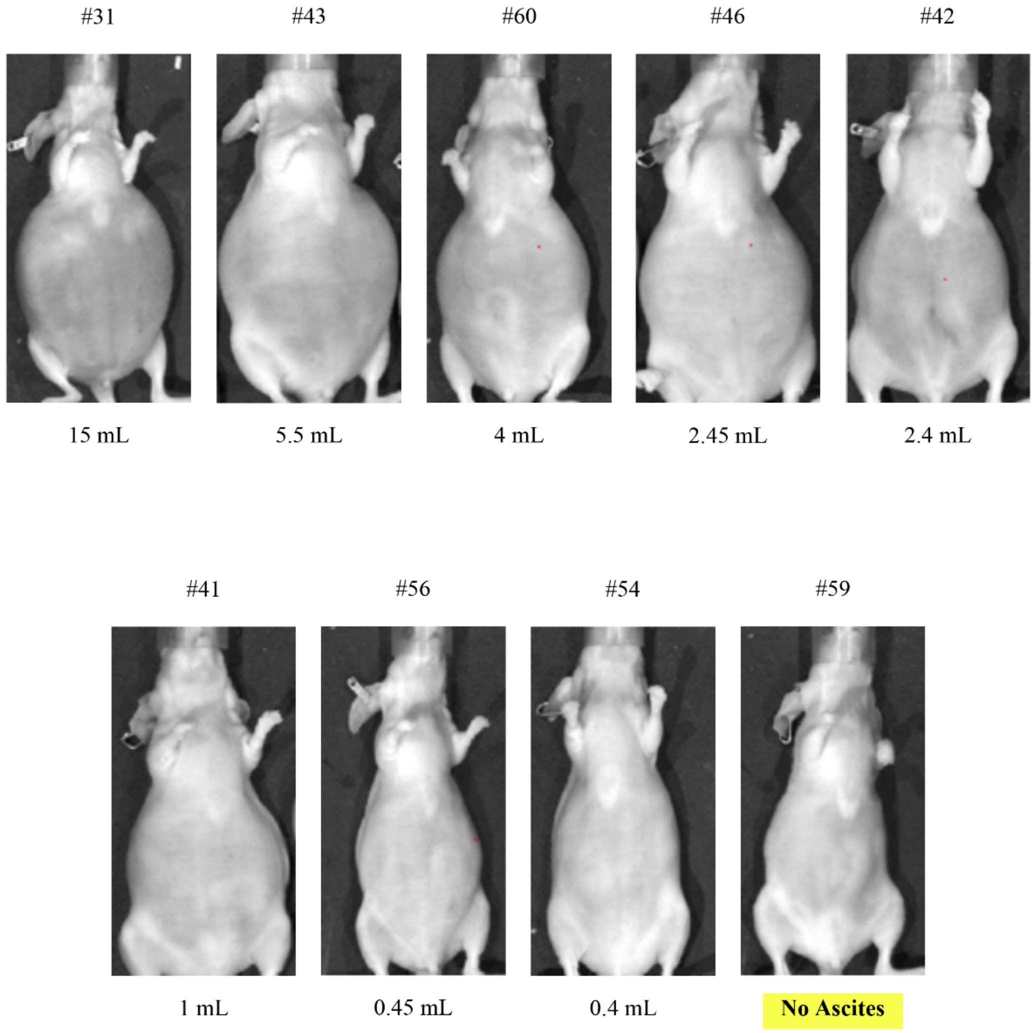

Supplementary Figure 2. Oral Talazoparib treated mice at the endpoint and volume of collectable ascites fluid.

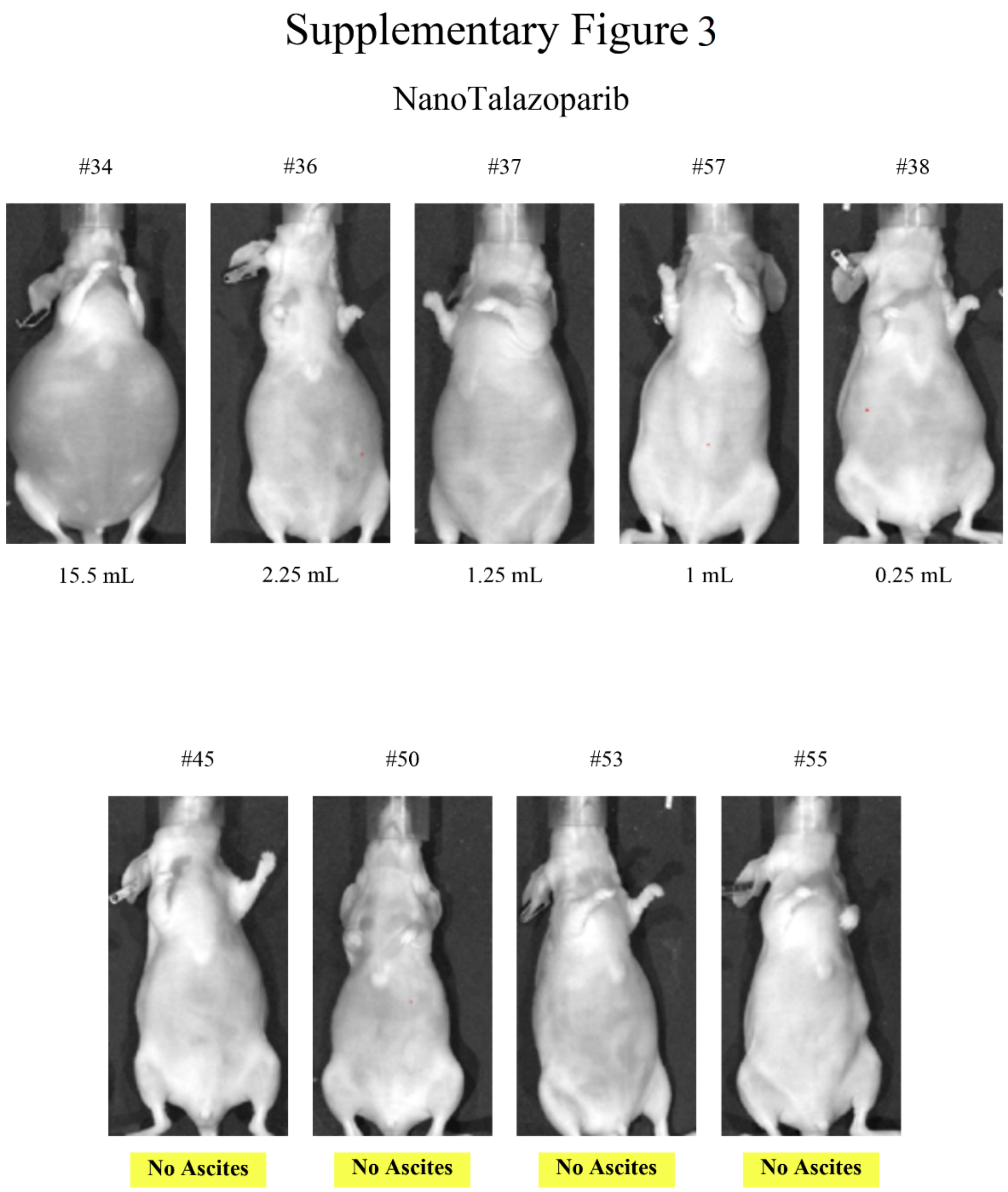

Supplementary Figure 3. NanoTalazoparib treated mice at the endpoint and volume of collectable ascites fluid.

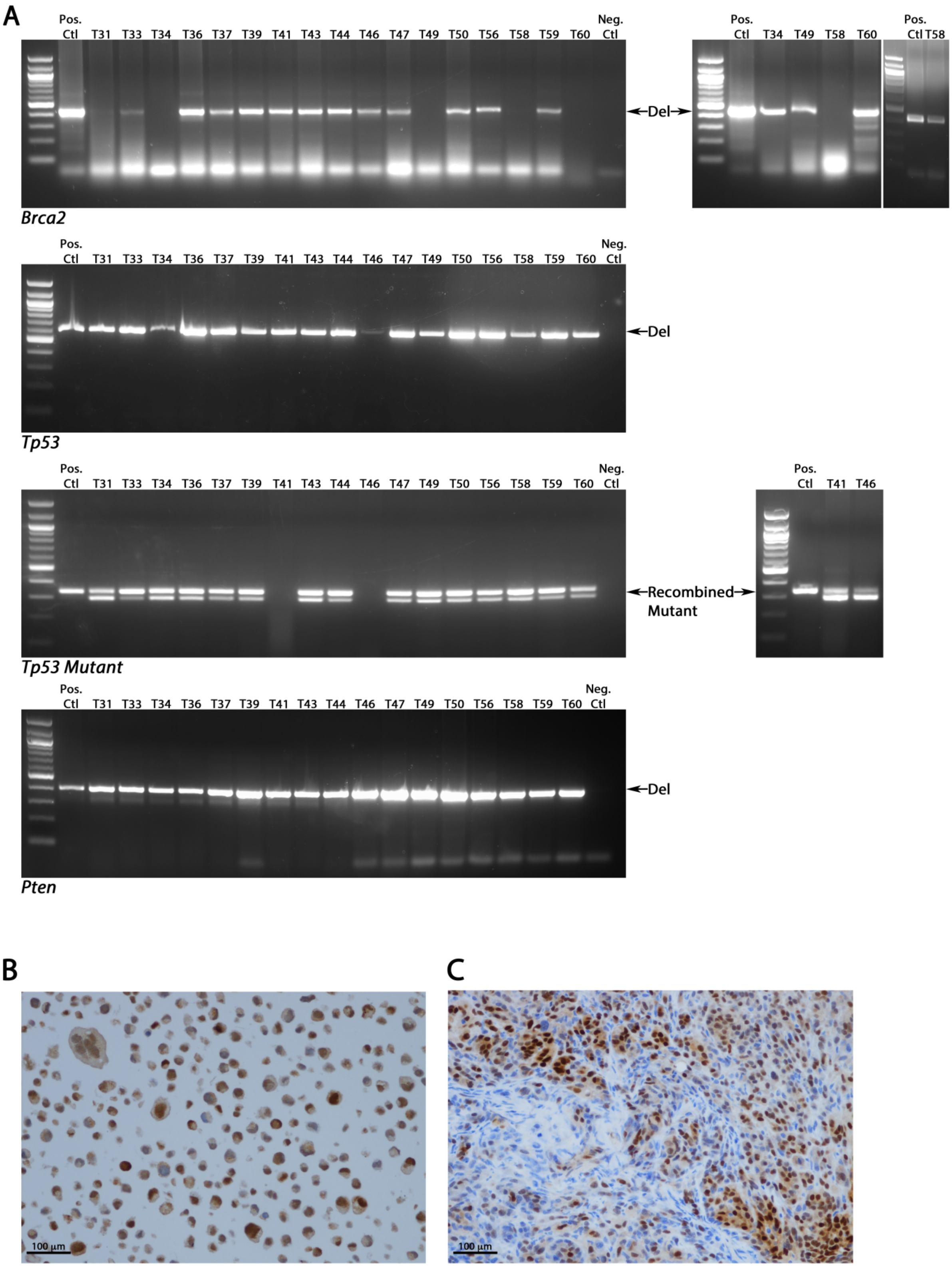
A
Pos. Ctl T31 T33 T34 T36 T37 T39 T41 T43 T44 T46 T47 T49 T50 T56 T58 T59 T60 Neg. Ctl
Del
Pos. Ctl T34 T49 T58 T60
Pos. Ctl T58
Brca2
Pos. Ctl T31 T33 T34 T36 T37 T39 T41 T43 T44 T46 T47 T49 T50 T56 T58 T59 T60 Neg. Ctl
Del
Tp53
Pos. Ctl T31 T33 T34 T36 T37 T39 T41 T43 T44 T46 T47 T49 T50 T56 T58 T59 T60 Neg. Ctl
Recombined
Mutant
Pos. Ctl T41 T46
Tp53 Mutant
Pos. Ctl T31 T33 T34 T36 T37 T39 T41 T43 T44 T46 T47 T49 T50 T56 T58 T59 T60 Neg. Ctl
Del
Pten
B
100 μm
C
100 μm

Supplementary Figure 4. Cre-mediated recombination PCRs for *Brca2*, *Tp53*, *Tp53 R172H mutant*, and *Pten* in murine xenograft tumors (A). PCRs for individual tumors required reaction optimization and due to a limited amount of tumor DNA for some samples not all reactions could be run on a single gel. Positive PAX8 immunohistochemical staining for the parental 3666 mFT tumor line (B), and tumor xenograft #58 (C). Images of PAX8 immunohistochemistry were taken at 20x magnification.
